# Dangers of including outcome at baseline as a covariate in latent change score models: Results from simulations and empirical re-analyses

**DOI:** 10.1016/j.heliyon.2023.e15746

**Published:** 2023-04-24

**Authors:** Kimmo Sorjonen, Michael Ingre, Gustav Nilsonne, Bo Melin

**Affiliations:** a Department of Clinical Neuroscience, Karolinska Institutet, Stockholm, Sweden; b Department of Gastroenterology and Hepatology, Karolinska University Hospital Huddinge, Karolinska Institutet, Stockholm, Sweden; c Institute for Globally Distributed Open Research and Education (IGDORE), Stockholm, Sweden; d Department of Psychology, Stockholm University, Stockholm, Sweden; e Meta-Research Innovations Center (METRICS), Stanford University, Palo Alto, USA

**Keywords:** Adjustment for baseline, Latent change score modeling, Matrix reasoning, Mutualism theory of cognitive development, Re-analyses, Regression to the mean, Vocabulary

## Abstract

Latent change score modeling is a type of structural equation modeling used for estimating change over time. Often change is regressed on the initial value of the outcome variable. However, similarly to other regression analyses, this procedure may be susceptible to regression to the mean. The present study employed simulations as well as re-analyses of previously published data, claimed to indicate reciprocal promoting effects of vocabulary and matrix reasoning on each other's longitudinal development. Both in simulations and empirical re-analyses, when adjusting for initial value on the outcome, latent change score modeling tended to indicate an effect of a predictor on the change in an outcome even when no change had taken place. Furthermore, analyses tended to indicate a paradoxical effect on change both forward and backward in time. We conclude that results from latent change score modeling are susceptible to regression to the mean when adjusting for the initial value on the outcome. Researchers are recommended not to regress change on the initial value included in the calculation of the change score when employing latent change score modeling but, instead, to define this parameter as a covariance.

## 1 Introduction

Regression to the mean is a phenomenon where extreme measures tend to change toward the mean [1]. If a person receives an unexpectedly high score, we should expect to observe a lower score on a subsequent occasion, and vice versa. What can be considered an unexpectedly high or low score differs between individuals. If a professional and a beginner dart player both receive a score of 15 (assuming that the maximum score is 3 × 10 = 30 and that the expected, i.e. mean, score for professionals and for amateurs is 26 and 16, respectively), we should expect a larger improvement to the next round for the professional than for the beginner. Conversely, if they both receive a score of 27, we can expect a larger decrease to the next round for the beginner than for the professional. Regression to the mean also functions backward in time. The beginner probably improved more from an earlier round to the round with 27 points while the professional probably decreased more from an earlier round to the round with 15 points. It is important to note that these predictions based on regression to the mean apply even without any change in the individuals’ true dart ability between the rounds.

Including a variable as a covariate in analyses is the statistical equivalent to assuming that entities/individuals have the same value on this variable. Consequently, if we were to include the dart score on a certain round as a covariate in analyses, we could expect, due to regression to the mean, a positive association between professionalism (vs. amateurism) and change in the score to a subsequent round. As the subsequent value is fully defined by the initial value and the change (subsequent value = initial value + change), given that the score on a certain round is included as a covariate in the model, the association between professionalism and the score on a subsequent round will be exactly the same as the association between professionalism and change in the score from the initial to the subsequent round. In standard regression notation, the coefficient β_1_ will be exactly the same if predicting.

E|Y_2_| = β_0_ + β_1_X + β_2_Y_1_ as when predicting.

E|Y_2_ – Y_1_| = β_0_ + β_1_X + β_2_Y_1_ [2].

Therefore, the predictions, based on regression to the mean and independent of any true change in dart ability, can be rephrased: When including the dart score on a certain round as a covariate, we can expect a positive association between professionalism and the score on a subsequent, or on an earlier, round.

If initial dart score is not included as a covariate in the analyses, predictions based on regression to the mean no longer apply. The expected differences in dart score between professionals and amateurs on two randomly selected rounds are the same:

$$E\left|score1_{pro}\;–\;score1_{ama}\right|=E\left|score2_{pro}\;–\;score2_{ama}\right|=E\left|{score}_{pro}\right|–\;E\left|{score}_{ama}\right|\text{,}$$

Hence, the expected change in score between two randomly selected rounds is the same for professionals and for amateurs,

$$E\left|score2_{pro}\;–\;score1_{pro}\right|=E\left|score2_{ama}\;–\;score1_{ama}\right|=0$$

Consequently, if the score on a certain round is not included as a covariate, no association between professionalism and change in the score from a certain round to a subsequent round is predicted due to regression to the mean. Therefore, unadjusted effects of a predictor, e.g. professionalism, on a change score on the dependent variable, e.g. dart ability measured as dart score, tend to be less affected by regression to the mean and give less biased estimates of the true association between the predictor and change on the dependent variable. This holds if, as in our example, there is an association between the predictor and the general level of the dependent variable, and if the dependent variable is measured with error [[3], [4], [5], [6]].

Latent change score modeling is a form of structural equation modeling for analyzing change between measurements [[7], [8], [9]]. A subsequently measured value on the outcome variable, Y_2_, is regressed on the initial value, Y_1_, and a latent change factor, ΔY, where both regression weights are fixed to 1, i.e. we define that.

Y_2_ = Y_1_ + ΔY and, consequently, that.

**Fig. 1:**
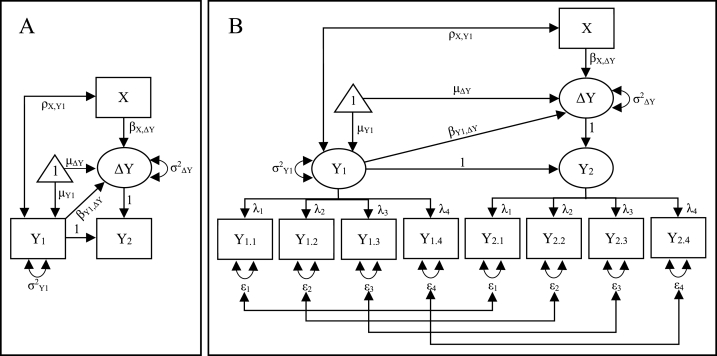
Illustration of latent change score modelling when the outcome Y is measured with a single item on two occasions (A) and when the outcome is measured with multiple items on two occasions (B). See the text for descriptions of the parameters.

ΔY = Y_2_ – Y_1_ (Fig. 1, panel A).

Other key features are that while the variances in Y_1_ and ΔY are usually freely estimated (σ^2^_Y1_ and σ^2^_ΔY_, respectively), the (error) variance in Y_2_ is set to zero, i.e. Y_2_ is fully defined by Y_1_ and ΔY. Similarly, the intercepts (the triangle in Fig. 1, panel A), corresponding to the means of Y_1_ and ΔY (μ_Y1_ and μ_ΔY_, respectively) are freely estimated, while the intercept is set to zero for Y_2_, meaning, in accordance with its definition, that Y_2_ is assumed to be zero if both Y_1_ and ΔY are zero. Furthermore, it is a common practice to regress the latent change score, ΔY, on the initial value of the outcome, Y_1_ (β_Y1,ΔY_). The model can be extended by regressing ΔY on some predictor X (β_X,ΔY_) in order to evaluate if X can predict change in Y between the measurements. In the present case, a covariance between X and Y_1_ (ρ_X,Y1_) is assumed.

If the outcome variable Y is measured with several indicators, Y_1_ and Y_2_ can be defined as latent variables (Fig. 1, panel B) [8,9]. In this case we wish to achieve measurement invariance, i.e. that a possible difference between Y_1_ and Y_2_ and, consequently, a value on ΔY that differs from zero, indicates a difference in degree and not that Y_1_ and Y_2_ measure different constructs [10,11]. To impose measurement invariance we can (1) fix the factor loadings of the same item to be equal at different measurements, e.g. the factor loading of Y_1.1_ and Y_2.1_, λ_1_, are fixed to equality; (2) fix unexplained residual variance of the same item to equality across measurements, e.g. the residual variance of Y_1.1_ and Y_2.1_, ε_1_; (3) fix the intercept of the same item to equality across measurements, e.g. the intercept of Y_1.1_ and Y_2.1_ (not shown in Fig. 1, panel B) [8,11]. Additionally, it is common to allow correlations between the error terms of the same item across measurements in order to account for item-specific residuals [8,12].

The use of latent change score models, rather than simpler regression models, has been recommended, e.g. by Kievit et al. [8], who suggest that latent change score models are flexible and allow good alignment between theoretical predictions and statistical estimands. However, latent change scores in single item models (see Fig. 1, panel A) are not free of measurement error [3]. We have previously shown that latent change score models, similarly as simpler regression models, are susceptible to regression to the mean and indicate an effect of a predictor, X, on the latent change score, ΔY, even with no true change in the outcome, Y [13,14]. However, to the best of our knowledge, this is the first time this bias in latent change score models is examined with controlled simulations. Moreover, we believe this is the first time the bias is examined in models with multiple indicators of the outcome variable. We also tested for the presence of bias in real-world data, using a dataset on the development of cognitive abilities previously analyzed with latent change score modeling [15].

## 2 Method

### 2.1 Simulations

**Fig. 2:**
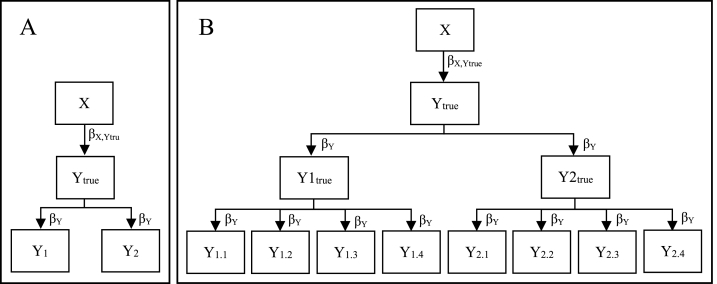
Illustration of data generation with no true change in the outcome, Y, between measurements, when the outcome is measured with a single item (A) and multiple items (B) on each occasion. Y1_true_ and Y2_true_ in panel B correspond to Y_1_ and Y_2_ in panel A, respectively. See the text for descriptions of the parameters.

Virtual participants (*N* = 10,000) were allocated an X-score from a random normal distribution (Fig. 2). We used large samples to minimize the influence of random fluctuation around the true population parameter value seen in small samples. Then the virtual participants were allocated a Y_true_-score from a random normal distribution with a defined population correlation with the X-score, β_X,Ytrue_. The correlation was set to all values between 0 and 1 in steps of 0.01, i.e. one hundred and one different values were used. In the next step, participants were allocated Y_1_ and Y_2_ scores, corresponding to observed values on the outcome at two different occasions. The population correlation between Y_true_ and Y_1_/Y_2_, β_Y_, was set to either 0.5, 0.7, or 0.9 (same for both), corresponding to the square root of the test-retest reliability in the measurement of the outcome, i.e. the simulations included situations where the test-retest correlation between Y_1_ and Y_2_ was equal to 0.5^2^ = 0.25, 0.7^2^ = 0.49, and 0.9^2^ = 0.81, respectively. In the simulations with multiple indicators (Fig. 2, panel B), in an additional step, participants were allocated scores on four indicators of Y_1_ and four indicators of Y_2_, respectively. The population correlation between Y_1_ and Y_2_ and their indicators, β_Y_, was set to the same value, i.e. 0.5, 0.7, or 0.9, as the correlation between Y_true_ and Y_1_ and Y_2_.

### 2.2 Empirical re-analyses

We re-analyzed data used by Kievit et al. [15], who collected data on matrix reasoning and vocabulary from 563 adolescents and young adults on two occasions, on average 1.48 years apart (an additional 221 individuals were measured at the first occasion). Measures were conducted with the Wechsler Abbreviated Scale of Intelligence (WASI-II). Kievit et al. [15] used latent change score modeling and found that high initial vocabulary predicted a larger increase in the matrix reasoning score between the occasions and that a high initial matrix reasoning score predicted a larger increase in vocabulary. They concluded that their finding supported the mutualism theory of cognitive development, according to which basic cognitive abilities reciprocally facilitate (causally) each other's longitudinal development [16]. Kievit et al. [15] have made their data and scripts available at the Open Science Framework at https://osf.io/93m2z/.

However, in their analyses Kievit et al. [15] regressed the change score on the initial value of the same ability, i.e. change in matrix reasoning score was regressed on initial matrix reasoning score in addition to initial vocabulary and change in vocabulary was regressed on initial vocabulary in addition to initial matrix reasoning score. Given the positive association between matrix reasoning scores and vocabulary, we suspect that this procedure may have made the findings by Kievit et al. [15] susceptible to regression to the mean.

### 2.3 Statistical analyses

Data, both simulated and empirical, were analyzed with latent change score models where the latent change score, ΔY, was regressed on a predictor, X, as described above and illustrated in Fig. 1 (further illustrations in the Result section). The main manipulation was to either regress the latent change score on the initial value on the outcome variable, i.e. including the parameter β_Y1,ΔY_ in the model (Fig. 1), or not. In the latter case, the regression effect was replaced by a covariance. We also conducted analyses where Y_1_ and Y_2_ changed positions in the models (see Fig. 1 and the Result section). In this case, the latent change score corresponds to change backward in time, i.e. how much the outcome, Y, has decreased from the first to the second measurement. The effect of the predictor, X, on this backward change should have an opposite sign compared with the effect on forward change, otherwise the effect seems to be due to regression to the mean. In summary, we estimated the effect of X on the latent change score of Y in eight different models (for each combination of single/multiple items, with/without adjustment for the initial score on the outcome, and forward/backward prediction) in 303 different groups (for each combination of 101 correlations between X and Y_true_ and three correlations between Y_true_ and Y_1_/Y_2_, *N* = 10,000 in each group). This resulted in 303 × 8 = 2424 separate estimations of the effect of X on the latent change score of Y. Simulations and analyses were conducted with R 4.1.0 statistical software [17] employing the lavaan package [18]. Scripts, which also generate the simulated data, are available at the Open Science Framework at https://osf.io/pw8ym/.

## 3 Results

### 3.1 Simulations

**Fig. 3:**
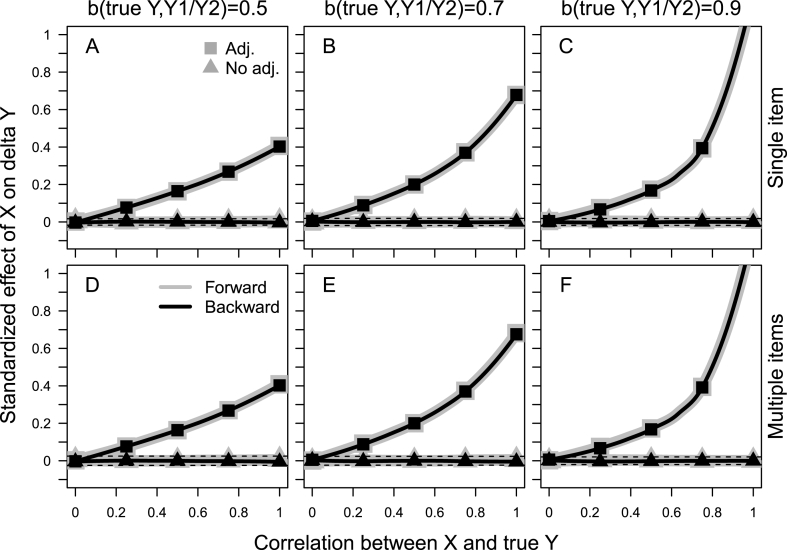
Standardized effect of a predictor X on the latent change score, ΔY, as a function of the correlation between X and Y_true_, in a situation with no true change in Y taking place. Separately for situations when the population correlation between Y_true_ and the initial and subsequent measurement of the outcome, Y_1_ and Y_2_ (as well as between Y_1_ and Y_2_ and their indicators in the second row), equals 0.5 (first column), 0.7 (second column), and 0.9 (third column). Data were generated according to the models in Fig. 2 (panel A for the first row and panel B for the second row), with no true change in Y taking place. Effects of X on ΔY have been calculated while adjusting for the initial value on Y (squares as markers) or when defining the association between Y_1_ and ΔY as a covariance (triangles as markers). ΔY captures latent change either forward in time, i.e. increase from Y_1_ to Y_2_ (thick gray lines), or backward in time, i.e. decrease from Y_1_ to Y_2_ (thinner black lines). The dashed horizontal lines indicate boundaries for a significant (p < .05) effect of X on ΔY with N = 10,000.

When data were generated as in Fig. 2 there was no true change in the outcome, Y, and a significant effect of X on the latent change in Y, β_X,ΔY_, should be viewed as spurious. The likelihood for spurious findings was high (Fig. 3), especially with a strong association between X and Y_true_ (high value on the X-axis) and when the effect of X on the latent change score, ΔY, was calculated while adjusting for the initial value on the outcome, Y_1_ (lines with squares as markers). When adjusting for the initial value of the outcome, the risk for spurious findings was not alleviated by a strong association between Y_true_ and Y_1_/Y_2_ (and between Y_1_ and Y_2_ and their indicators in the simulations with multiple items, second row in Fig. 3), i.e. high reliability in the measurement of the outcome (the lines with squares as markers remain high in panels C and F). With both a very strong correlation between X and Y_true_ and a high reliability in the measurement of the outcome, Y, the standardized effect of X on the latent change score, ΔY, tended even to be above one (right part of panels C and F). It is interesting to note that the effect of X on ΔY was the same when ΔY represented increase in Y from the initial to the subsequent measurement (gray lines) and when it represented increase in Y backward in time from the subsequent to the initial measurement, i.e. decrease from the first to the second measurement (black lines).

The likelihood for spurious findings was much lower when not adjusting the effect of X on ΔY for the initial value on the outcome, i.e. when the regression effect of Y_1_ on ΔY was replaced by a covariance (the lines with triangles as markers in Fig. 3). All results were identical when data were generated and analyzed with a single indicator of Y_1_ and Y_2_ (as in panel A in Fig. 1, Fig. 2) and with multiple indicators of Y_1_ and Y_2_ (as in panel B in Fig. 1, Fig. 2), respectively.

### 3.2 Empirical re-analyses

**Fig. 4:**
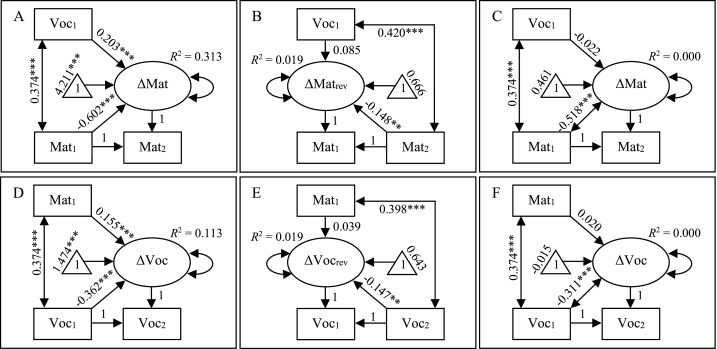
Latent change score models with change in matrix reasoning score predicted from initial vocabulary (panels A, B, and C) and change in vocabulary predicted from initial matrix reasoning score (panels D, E, and F) when including the initial value on the outcome ability as a covariate (panels A and D), when predicting change backward in time and including the subsequent value on the outcome ability as a covariate (panels B and E), and when not including initial value on the outcome as a covariate (i.e. replacing the regression effect by a covariance, panels C and F). Standardized coefficients. *p < .05, **p < .01, ***p < .001.

Re-analyses of data from Kievit et al. [15] indicated, as in the original study, that when including initial value on the outcome ability as a covariate, a high initial vocabulary predicted a larger increase in the matrix reasoning score between measurement occasions and a high initial matrix reasoning score predicted a larger increase in vocabulary (Fig. 4, panels A and D). However, when predicting change backward in time and conditioning on the subsequent value on the outcome ability, the effects did not change sign, as would have been expected with true causal effects. Although weak and non-significant, the effects remained positive (Fig. 4, panels B and E). This means that here, with the same data, a high initial vocabulary predicted (non-significantly) a slightly smaller increase in the matrix reasoning score and a high initial matrix reasoning score predicted (non-significantly) a slightly smaller increase in vocabulary. When change was not conditioned on the initial value on the outcome ability, initial vocabulary and initial matrix reasoning score had hardly any effect on change in the other ability (Fig. 4, panels C and F). Note that the only difference between the models in panels A and D and the models in panels C and F, respectively, is that in the latter the association between initial value and change in the outcome ability is defined as a covariance rather than as a regression effect.

It may seem that the models in panels A and C in Fig. 4 make very different predictions of the association between initial vocabulary and change in matrix reasoning score, with a significant positive and a non-significant negative effect, respectively. However, the effect in panel A is positive only because it has been estimated without regard for the association between initial vocabulary and initial matrix reasoning score. When accounting for this association, the total effect of initial vocabulary on change in matrix reasoning score in the model in panel A is 0.203 + 0.374 × −0.602 = −0.022, i.e. exactly the same as the effect in the model in panel C. Similarly, when accounting for the positive association between initial vocabulary and subsequent matrix reasoning score in the model in panel B, the total effect of initial vocabulary on the backward change in matrix reasoning score is 0.085 + 0.420 × −0.148 = 0.023, which indicates a decrease by 0.023 from the initial to the subsequent measurement (difference from 0.022 due to rounding). The same logic applies to the effects of initial matrix reasoning score on change in vocabulary in the models in panels D–F.

## 4 Discussion

The present simulations showed that latent change score modeling is susceptible to regression to the mean when regressing change on the initial value on the outcome variable. With an association between a predictor, X, and the true value on the outcome variable, Y, findings can indicate that X has an association with change in Y even if no true change has taken place. This is in accordance with a similar effect demonstrated in simpler regression models [[3], [4], [5], [6]]. Additionally, the present simulations indicated that this problem might not be alleviated by high reliability in the measurement of the outcome or by measuring the outcome with several indicators and defining the outcome as a latent, rather than as an observed, variable. The risk for a spurious effect, due to regression to the mean, of X on the change in Y was no longer present if defining the association between initial Y and the change in Y as a covariance instead of as a regression effect.

In the empirical re-analyses, when including the initial value on the outcome variable as a covariate in the model, initial vocabulary and initial matrix reasoning had positive effects on change in matrix reasoning and on change in vocabulary between two measurement occasions, respectively. This is, not surprisingly, in line with the findings in the original study by Kievit et al. [15]. These authors concluded that the results supported the mutualism theory of cognitive development, according to which cognitive abilities promote the development of other cognitive abilities [16]. However, if reversing the latent change score factor, so that it captured change backward in time from the subsequent to the initial occasion, and conditioning change on the subsequent value on the outcome variable, the effects of initial vocabulary and initial matrix reasoning score on the change in matrix reasoning and vocabulary, respectively, remained positive (non-significant). This would mean that a high score on one ability predicts (non-significantly) a smaller increase on the other ability, in direct contradiction of the mutualism theory.

Surely, if analyses of the same data can be used to conclude both X and its exact opposite, the validity of either conclusion can be doubted. We propose that a more tenable description of the findings is that if conditioning on measured vocabulary (matrix reasoning) at a certain occasion we can, due to regression to the mean, expect a positive association between matrix reasoning score (vocabulary) and another measured value, either forward or backward in time, on vocabulary (matrix reasoning). The conclusion that observed adjusted associations probably are due to regression to the mean, rather than any true associations between initial/subsequent ability and change in the other ability, were supported by the fact that the association between initial vocabulary (matrix reasoning) and change in matrix reasoning (vocabulary) vanished if not adjusting for initial matrix reasoning (vocabulary).

### 4.1 Limitations

In all present simulations, the true population effect of predictor X on change in outcome Y was equal to zero. Consequently, the present findings should not be generalized to situations where a predictor has a true effect on change in an outcome.

In the simulations with multiple indicators, the observed scores were assumed to be indicators, measured with error, of true occasion-specific indicators, including occasion-specific error, of individuals’ true general value on the outcome variable (see panel B in Fig. 2). We believe this hypothetical situation to be realistic. The occasion-specific measurement errors could be due to factors such as temporary illness or stressful events (e.g. missing the bus on the way to the measurement location) or low motivation. Of course, other data-generating models, for example without occasion-specific measurement errors, are possible. However, this possibility does not invalidate the findings from the simulations analyzed here.

The findings in the present re-analyses of empirical data should not be interpreted as a conclusive refutation of the mutualism theory of cognitive development, but rather as a critique of the alleged evidence for the theory in Kievit et al. [15]. In a later study with three waves of data, Kievit et al. [19] employed latent growth modeling. Without adjustment for the initial value on the outcome variable, they found a positive association between predicted initial vocabulary (matrix reasoning score) and predicted individual slopes in matrix reasoning (vocabulary) across the three measurements. These findings should be less susceptible to the influence of regression to the mean.

### 4.2 Conclusions

As in simpler regression models, if including outcome at baseline as a covariate in latent change score models, the effect of a predictor, X, on the latent change in the outcome variable, Y, is susceptible to regression to the mean. Given an association between X and the true value on Y, the analyses may indicate an effect of X on the change in Y even if no true change has taken place. Consequently, we recommend researchers not to regress change on the initial value on the outcome variable when conducting latent change score modeling but, instead, to define this parameter as a covariance. If the effect of initial outcome on change is judged as important to retain in the model for some reason, researchers can confirm the logic of their conclusions by reversing the latent change factor so it captures change from the subsequent to the initial measurement. If not due to regression to the mean, this reversal of change should reverse the sign of the effect of X on the change in Y.

## Author contribution statement

Kimmo Sorjonen: Conceived and designed the experiments; Performed the experiments; Analyzed and interpreted the data; Contributed reagents, materials, analysis tools or data; Wrote the paper.

Michael Ingre: Conceived and designed the experiments; Analyzed and interpreted the data; Wrote the paper.

Gustav Nilsonne: Conceived and designed the experiments; Analyzed and interpreted the data; Wrote the paper

Bo Melin: Conceived and designed the experiments; Analyzed and interpreted the data; Wrote the paper.

## Data availability statement

Scripts, which also generate the simulated data, are available at the Open Science Framework at https://osf.io/pw8ym/. Data used in the re-analyses are also available at the Open Science Framework, at https://osf.io/93m2z/ (Kievit et al., 2017).

## Declaration of interest statement

The authors declare no conflict of interest.
